# Revealing hidden moves: *Staphylococcus* spp. exhibit motility in semisolid media

**DOI:** 10.1128/spectrum.02620-25

**Published:** 2025-11-06

**Authors:** Jessica F. S. Lopes, Thiago F. S. Coimbra, Thais Glatthardt, Clara Maria Guimarães Silva, Kátia Regina Netto dos Santos, Bruno A. Penna, Rosana B. R. Ferreira

**Affiliations:** 1 Instituto de Microbiologia Paulo de Góes, Universidade Federal do Rio de Janeiro 28125, Rio de Janeiro, Brazil; 2 Department of Physiology and Pharmacology, Health Research Innovation Centre, University of Calgary 198999, Calgary, Alberta, Canada; 3 Universidade Federal Fluminense 28110, Niterói, Brazil; 4 Department of Molecular Biosciences, The University of Kansas 4202, Lawrence, Kansas, USA; The University of Texas at Austin, Austin, Texas, USA

**Keywords:** *Staphylococcus*, *S. pseudintermedius*, bacterial motility, *S. schleiferi*, spreading, veterinary microbiology

## Abstract

*Staphylococcus* spp. are generally considered non-motile due to the absence of active motility structures. However, *Staphylococcus aureus*, *Staphylococcus xylosus*, and *Staphylococcus epidermidis* have demonstrated passive movement. This study investigated the motility of eight additional *Staphylococcus* species in semisolid media. While most species exhibited some motility, *Staphylococcus pseudintermedius* and *Staphylococcus schleiferi* displayed significantly higher levels of motility. Then, we further evaluated the motility of 49 *S*. *pseudintermedius* and 30 *S*. *schleiferi* isolates, revealing strain-dependent variations. The influence of glucose, NaCl, temperature (35 and 39°C), and culture media (tryptic soy broth [TSB], brain heart infusion [BHI], and Luria Bertani [LB]) on the motility of these two species was evaluated. Glucose and NaCl induced morphological changes in the *S. pseudintermedius* colonies. In *S. schleiferi*, NaCl decreased, while glucose increased motility. Temperature and culture media also significantly affected motility, with *S. schleiferi* showing reduced movement in BHI and both species exhibiting decreased motility in LB compared to TSB. Shedding light on surface motility in *Staphylococcus*, an understudied phenomenon, can provide insights to elucidate their biology and clarify their mechanism of colonization, survival, and pathogenesis.

**IMPORTANCE:**

The ability to move is an important characteristic for many bacteria to colonize and infect hosts, yet motility in *Staphylococcus* is understudied. Our work reveals that several species, notably *S. pseudintermedius* and *S. schleiferi*, can actively move across surfaces. This previously hidden capability may be crucial for how these bacteria spread and cause infections. Our evaluation of how different growth conditions and specific genes impact this trait shows species-dependent effects. This study opens new research avenues for understanding the mechanisms these bacteria use to spread within both the environment and their hosts.

## INTRODUCTION

The ability of bacteria to move can be crucial for their survival and dissemination, as this trait allows access to different environments for obtaining vital resources. For pathogens, it is also an important virulence factor, playing a fundamental role in the success of infections (1). Bacteria move through a variety of mechanisms, including the use of structures, such as extracellular appendages, or even complex chemosensory systems (1, 2). Bacterial motility is classified as either active or passive. Active movements rely on energy-dependent cellular mechanisms, giving bacteria control over their direction, whereas passive movement requires environmental modifications to generate the force needed to propel the cells (2).

*Staphylococcus* species are traditionally considered non-motile primarily due to their lack of extracellular appendages, such as flagella and pili, which aid in locomotion (3). However, passive motility has been described in a few species, including *Staphylococcus epidermidis*, *Staphylococcus aureus*, and *Staphylococcus xylosus* (4–6). In *S. aureus*, this phenotype is better understood, and motility is known to be directly associated with the accessory gene regulator (*agr*), a quorum-sensing system responsible for regulating virulence genes in *Staphylococcus* (7). The motility observed in *S. aureus* has been called spreading, a passive mechanism involving the production of phenol-soluble modulins (PSMs), which may act as surfactants enabling the bacteria to disperse individually (2).

Besides surfactant production, other factors have been shown to be required for *S. aureus* spreading (2). These include genes, such as *tagO*, *ypfP*, and the *dlt* operon, which are involved in teichoic acid synthesis (6), *nuc1* and *nuc2*, which encode extracellular nucleases (8), and *hemY* and *ctaA*, genes responsible for the production of heme A synthase and protoporphyrinogen oxidase, respectively (9). The addition of glucose, glycerol, fructose, and other sugars has also been reported to stimulate the *S. aureus* motility (10). On the contrary, Sortase A (encoded by the *srtA* gene), a cell surface export protein, has been described as a repressor of spreading (11).

Currently, the genus *Staphylococcus* contains over 70 described species, some of which are considered members of the skin microbiome of humans and other mammals, such as dogs (12, 13). Some of these species, including *S. capitis*, *S. epidermidis*, *S. haemolyticus*, *S. hominis*, *S. lugdunensis*, *S. saprophyticus*, *and S. warneri*, can be frequently associated with opportunistic infections in humans, especially in people with an immunodeficiency (14). *S. pseudintermedius* and *S. schleiferi* are species more commonly found in dogs, with a significant impact on veterinary medicine due to their involvement in infections, such as pyoderma and otitis in dogs (15–17). Occasionally, these species can also be isolated from infections in immunocompetent humans (18, 19).

Despite earlier studies on *S. aureus*, *S. epidermidis*, and *S. xylosus* motility (4–6), no studies have reported motility in other *Staphylococcus* species commonly associated with opportunistic infections in humans and other animals. In this context, this study aimed to investigate if other *Staphylococcus* species present motility on semisolid media, evaluate whether growth conditions can affect their motility, and identify genes that may influence the motility level. A better understanding of these aspects could provide new insights into the biology of these microorganisms, with possible implications for understanding their colonization, survival, and pathogenicity.

## MATERIALS AND METHODS

### Bacterial strains

A total of 105 previously identified strains were analyzed, comprising *S. capitis* (five strains), *S. haemolyticus* (four strains), *S. hominis* (four strains), *S. lugdunensis* (four strains), *S. pseudintermedius* (49 strains), *S. saprophyticus* (four strains), *S. schleiferi* (30 strains), and *S. warneri* (five strains). These strains were originally isolated from humans and other animals either from colonization or infection cases. Additionally, the experiments included two human-derived *S. aureus* isolates as positive and negative controls of motility (*S. aureus* 74 and *S. aureus* MU50 strain, respectively) (20–22).

### Motility assays in semisolid media

For the motility assays, the bacterial strains were initially plated on tryptic soy agar (TSA, Titan Biotech, India) and incubated at 37°C for 24 h. Then, a colony from each strain was inoculated into a test tube containing 3 mL of tryptic soy broth (TSB, Kasvi, Italy) and incubated again at 37°C for 24 h. The motility assays were performed following the protocol described by Kaito and Sekimizu (6), with some modifications. For the regular assays, two types of media were used: TSB containing 1.5% agar (Becton, Dickinson, USA) and TSB supplemented with 0.24% agar (Becton, Dickinson, USA). Both media were sterilized at 121°C for 15 min and stored at 55°C in a water bath until use. After 30 min, 8 mL of TSB with 1.5% agar was poured into Petri dishes (60 × 10 mm), forming the solid base required to support the semisolid medium. To allow solidification, the plates were left open in a laminar flow hood for 10 min. Subsequently, 2 mL of TSB supplemented with 0.24% agar was added on top of the solidified base. The plates were kept open in the laminar flow hood for an additional 10 min to allow the upper layer to solidify. Then, 2 µL of a bacterial suspension at an optical density (OD600nm) of 0.6 (approximately 1.5 × 10⁸ CFU/mL) was carefully inoculated into the center of the Petri dish, ensuring that the pipette tip did not touch the agar. After inoculation, the plates were left in the laminar flow hood for 10 min, sealed with parafilm, and incubated at 37°C for 18 h. After incubation, the colonies were photographed, and the diameter of the spreading was measured at its largest dimension using a ruler. Each experiment was performed in triplicate for each strain, and the average growth diameter was recorded.

### Motility level classification

To represent the variations observed with the different *S. pseudintermedius* and *S. schleiferi* strains, a motility level classification was established based on the motility results obtained with the positive and negative controls. The average diameter obtained with three biological replicates of the negative control (*S. aureus* Mu50) + 2 standard deviations was used as a cutoff for non-motile strains (8.3 mm), while the average diameter obtained with three biological replicates of the positive control (*S. aureus* 74) + 2 standard deviations was used as a cutoff for high motility strains (19.7 mm). The midway point between the values was obtained (14 mm) and used as the cutoff to separate low motility and medium motility strains.

### Motility assays in different culture media

Selected bacterial strains were initially cultured on either the brain heart infusion agar (BHI agar, Kasvi, Italy) or Luria Bertani medium (LB agar, Kasvi, Italy) and incubated at 37°C for 24 h. A colony from each strain was then inoculated into a test tube containing either 3 mL of BHI or LB broth and incubated at 37°C for 24 h. Subsequently, the procedures described in the motility assay in Section 2.1 were replicated using BHI agar as the solid base and brain heart infusion broth (BHI broth, Kasvi, Italy) supplemented with 0.24% agar (Becton, Dickinson, USA) on the solidified base or LB agar containing 1.5% agar as the base and LB broth supplemented with 0.24% agar for the upper layer.

### Motility assays in semisolid media at different temperatures

The procedures described in Section 2.1 were also followed to assess motility at different temperatures. After inoculation, the plates were placed in a laminar flow hood for 10 min, sealed with parafilm, and incubated at either 35 or 39°C for 18 h.

### Motility assays in the presence of glucose and NaCl

For the glucose assays, monohydrated dextrose (INLAB, Brazil) at 1% was added to the semisolid medium (TSB containing 0.24% agar) before being poured onto the solid base, which already contained 2.5 g/L of dextrose and 5.0 g/L of NaCl. For the NaCl assays, sodium chloride (P.A.; Isofar, Brazil) was added to the culture medium during the preparation of the semisolid medium (TSB containing 0.24% agar). After preparation and bacterial inoculation as described above, the plates were sealed with parafilm and incubated at 37°C for 18 h.

### Statistical analysis

The statistical comparisons in this study were performed using one-way analysis of variance (GraphPad Prism Software, San Diego, USA). Differences between experimental conditions were considered statistically significant when the *P*-value was less than 0.05.

## RESULTS AND DISCUSSION

### Evaluation of motility in semisolid media among different *Staphylococcus* species

**Fig 1:**
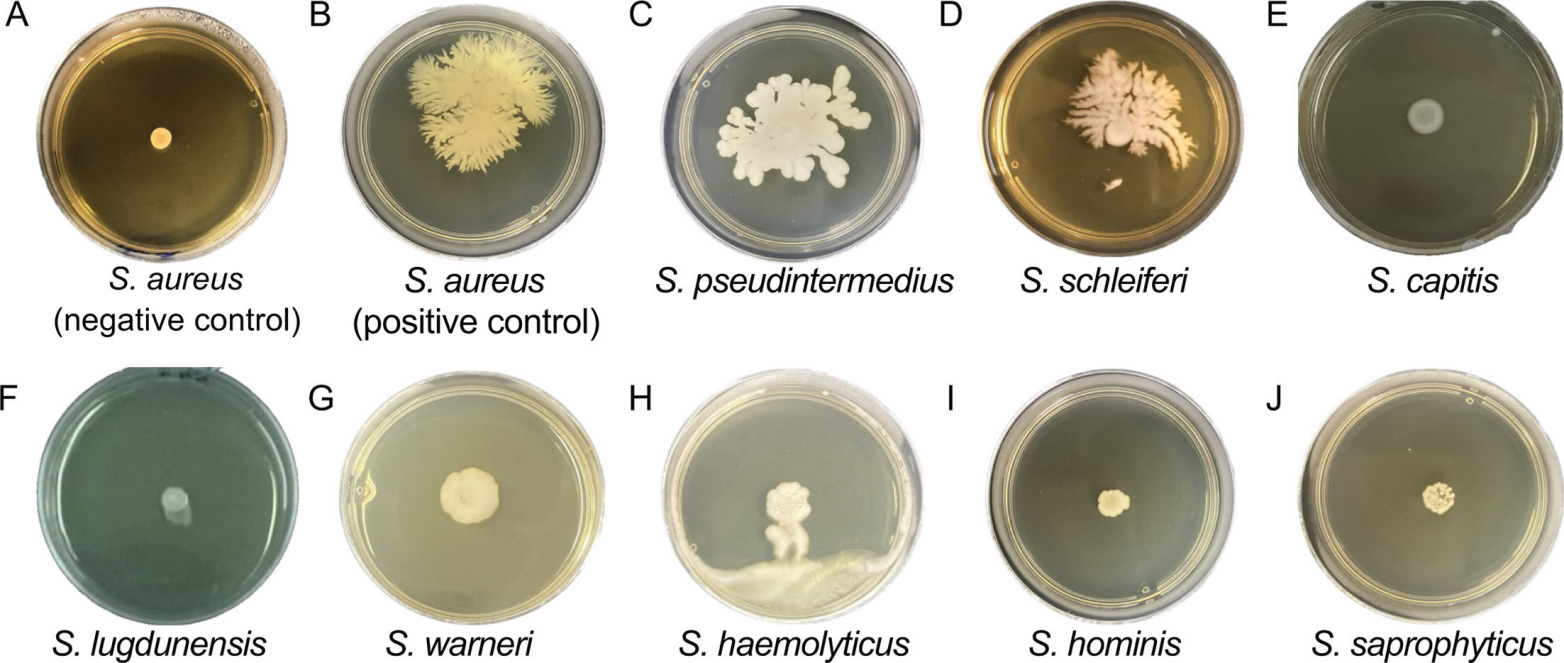
Spreading motility profiles of the representative strains of different *Staphylococcus* species in the semisolid TSB medium. (**A**) *S. aureus* Mu50 (negative control); (**B**) *S. aureus* 74 (positive control); (**C**) *S. pseudintermedius;* (**D**) *S. schleiferi*; (**E**) *S. capitis*; (**F**) *S. lugdunensis*; (**G**) *S. warneri*; (**H**) *S. haemolyticus*; (**I**) *S. hominis*; and (**J**) *S. saprophyticus*.

Motility is an adaptive trait related to the microbial colonization of various hosts and surfaces and is, therefore, closely linked to the survival and dissemination of certain bacterial species (2). The present study expands the current knowledge of this understudied trait in the *Staphylococcus* genus, which contains species critical to the health of both humans and animals (12). We started by investigating if certain *Staphylococcus* species could move on semisolid surfaces under the same conditions previously used to report *S. aureus* spreading motility (6). Initially, we screened for representative strains of nine *Staphylococcus* species for motility, including *S. capitis* (five strains), *S. haemolyticus* (four strains), *S. hominis* (five strains)*, S. lugdunensis* (four strains), *S. pseudintermedius* (12 strains), *S. saprophyticus* (four strains), *S. schleiferi* (four strains), and *S. warneri* (five strains) (Fig. 1; Table 1). *S. haemolyticus*, *S. hominis*, *and S. warneri* showed results that are not statistically significantly different from the negative control (Table 2), suggesting that these species are non-motile. *S. capitis*, *S. lugdunensis*, and *S. saprophyticus* showed a slightly higher motility compared to the negative control (*S. aureus* Mu50) but significantly lower than the positive control (*S. aureus* 74). Although we did not observe significant motility for these species in our study, it is certainly possible that other isolates from these species do have the ability to move on semisolid agar, and testing a larger number of strains is necessary to further investigate this. Nevertheless, our results indicated that *S. pseudintermedius* and *S. schleiferi* have displacement capacities similar to *S. aureus*, a fact previously unreported for these species.

**TABLE 1:** Average motility diameter of the different *Staphylococcus* species

Species	Number of strains evaluated	Average motility diameter in mm (*+*SD^*a*^)
*S. pseudintermedius*	12	20.4 (*+*7.4)
*S. schleiferi*	4	18.3 (*+*7.7)
*S. saprophyticus*	4	9 (*+*0.0)
*S. capitis*	5	8.5 (*+*0.7)
*S. lugdunensis*	4	8.5 (*+*1.3)
*S. haemolyticus*	4	8 (*+*2.0)
*S. warneri*	5	7.2 (*+*3.1)
*S. hominis*	4	6 (*+*0.6)
*S. aureus* (74—positive control)	1	16.1 (*+*1.8)
*S. aureus* (Mu 50—negative control)	1	5.7 (*+*1.3)

^*a*^ SD: standard deviation.

**TABLE 2:** Classification of motility among the *S. pseudintermedius* and *S. schleiferi* strains

Species	Level of motility	Number of strains	Average of diameter in mm
*S. pseudintermedius*	High	20 (40.8%)	≥19.7
	Medium	16 (32.6%)	14.1–19.6
	Low	13 (26.5%)	8.4–14
	Non-motile	0	≤8.3
*S. schleiferi*	High	9 (30%)	≥19.7
	Medium	9 (30%)	14.1–19.6
	Low	12 (40%)	8.4–14
	Non-motile	0	≤8.3

**Fig 2:**
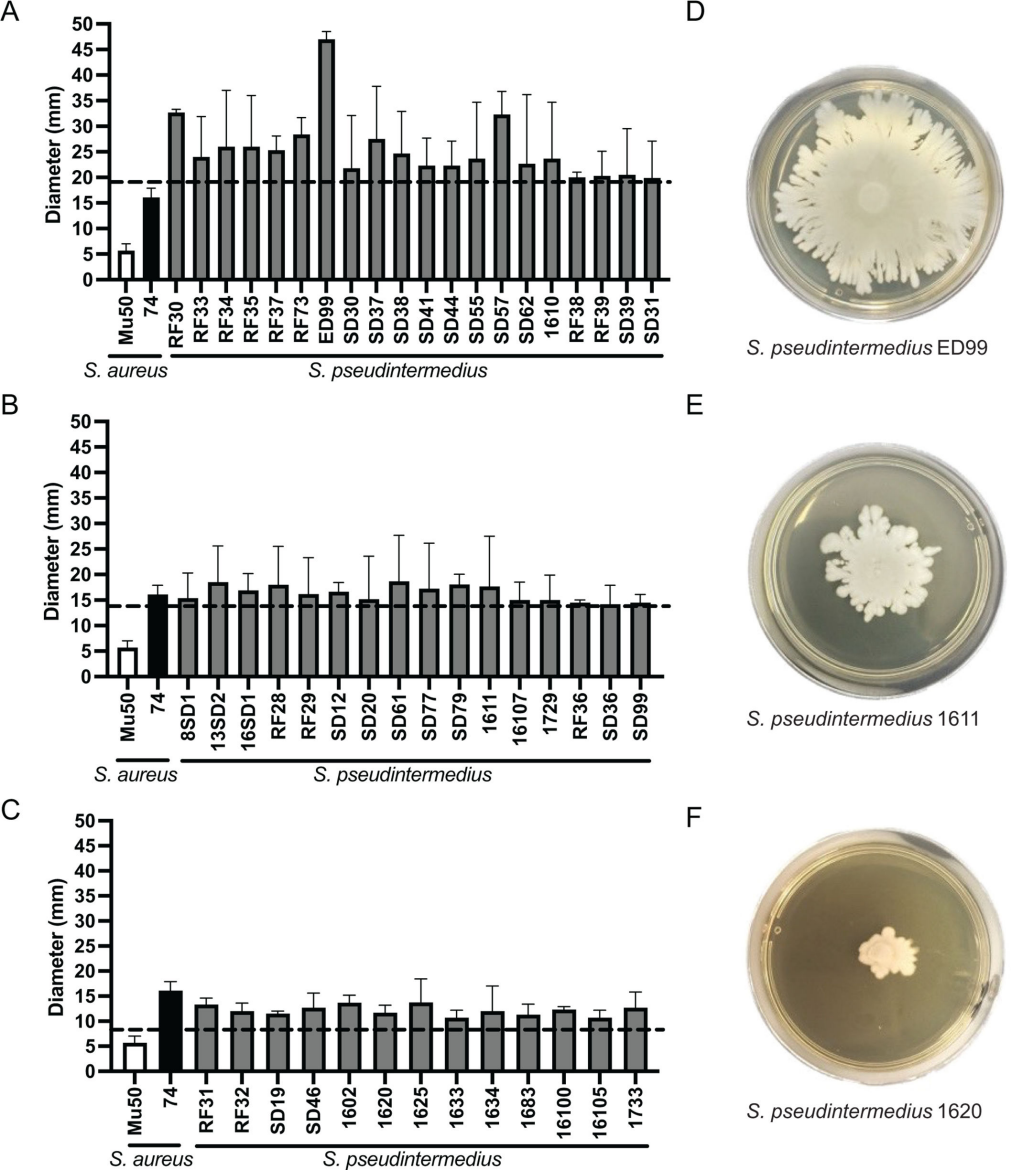
Motility of the *S. pseudintermedius* strains in the semisolid medium at different classification levels. (**A**) Diameter of the *S. pseudintermedius* strains classified as exhibiting high motility; (**B**) diameter of the *S. pseudintermedius* strains classified as exhibiting medium motility; (**C**) diameter of the *S. pseudintermedius* strains classified as exhibiting low motility; (**D**) *S. pseudintermedius* strain ED99 (representative of high motility); (**E**) *S. pseudintermedius* strain 1611 (representative of medium motility); and (**F**) *S. pseudintermedius* strain 1620 (representative of low motility). The dashed line indicates the diameter of motility used for each category: 19.7 mm for high motility, 14.1 mm for medium motility, and 8.4 mm for low motility.

To expand our initial findings obtained with 12 strains of *S. pseudintermedius*, we evaluated the spreading of a total of 49 *S*. *pseudintermedius* canine isolates on semisolid media. All the isolates exhibited motility, with variations in the diameter of the spreading, as illustrated in Fig. 2. Based on the motility level classification that represents the variations observed with the different strains, the average diameter obtained with the negative control (*S. aureus* Mu50) + 2 standard deviations was used as a cutoff for non-motile strains, while the average diameter obtained with the positive control (*S. aureus* 74) + 2 standard deviations was used as a cutoff for high motility strains. The midway point between the values was obtained (14 mm) and used as the cutoff to separate low- and medium-motility strains. Using the criteria we established (see Methods), 20 strains (40.8%) were classified as having high motility; 16 (32.6%) displayed moderate motility; and 13 (26.5%) exhibited low motility (Table 2). None of the strains were classified as non-motile, underscoring that this is a widespread phenomenon in this species.

**Fig 3:**
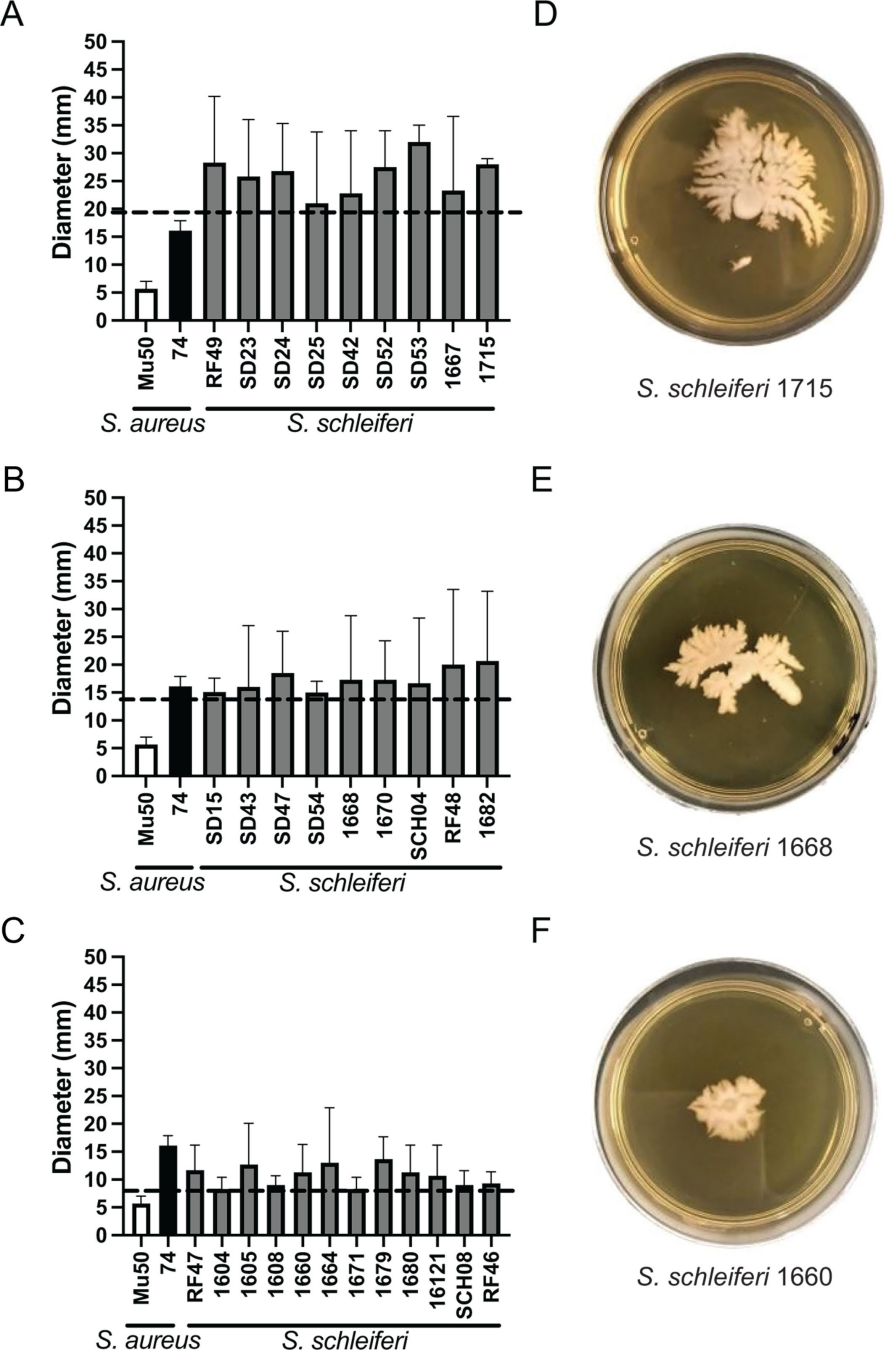
Motility of the *S. schleiferi* strains in the semisolid medium at different classification levels. (**A**) Diameter of the *S. schleiferi* strains classified as exhibiting high motility; (**B**) diameter of the *S. schleiferi* strains classified as exhibiting medium motility; (**C**) diameter of the *S. schleiferi* strains classified as exhibiting low motility; (**D**) *S. schleiferi* strain 1715 (representative of high motility); (**E**) *S. schleiferi* strain 1668 (representative of medium motility); and (**F**) *S. schleiferi* strain 1660 (representative of low motility). The dashed line indicates the diameter of motility used for each category: 19.7 mm for high motility, 14.1 mm for medium motility, and 8.4 mm for low motility.

To better explore our initial motility results obtained with four strains of *S. schleiferi*, the spreading of a total of 30 *S*. *schleiferi* canine isolates was also evaluated in semisolid media. Similar to what was observed with *S. pseudintermedius* strains, all *S. schleiferi* isolates were motile, but the level of motility was strain-dependent (Fig. 3). Among the *S. schleiferi* isolates, 30% (9 isolates) were classified as having high motility, 30% (9 isolates) as having moderate motility, 40% (12 isolates) as having low motility, and no strains being classified as non-motile (Table 2). Similar to what we observed with *S. pseudintermedius*, motility on semi-solid surfaces seems to be a common feature among *S. schleiferi* isolates.

### Impact of different growth conditions on *S. pseudintermedius* motility

**Fig 4:**
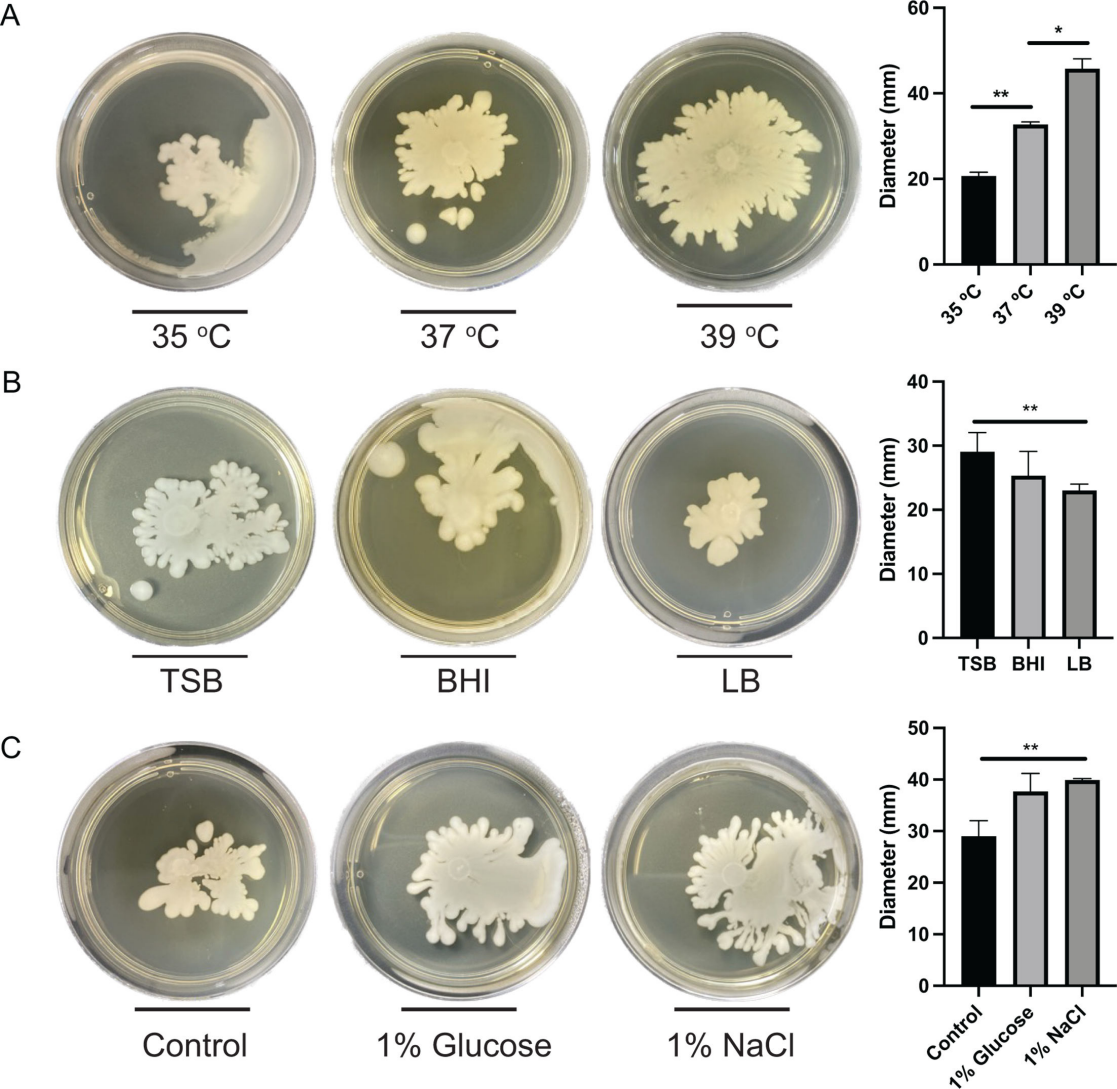
Impact of different growth conditions on *S. pseudintermedius* motility. (**A**) Evaluation of the *S. pseudintermedius* strain RF30 motility in the TSB semisolid media at 35, 37, and 39°C. (**B**) Evaluation of the *S. pseudintermedius* strain RF30 motility in the TSB, BHI, and LB semisolid media. (**C**) Evaluation of the *S. pseudintermedius* strain RF30 motility in the TSB semisolid media supplemented with either 1% glucose or 1% NaCl. *: *P* < 0.05; **: *P* < 0.01.

The standard motility assays described in the literature for *S. aureus* preconizes the use of TSB media supplemented with 0.24% of agar and incubation at 37°C for either 10 or 24 h depending on the protocol (6, 8, 22, 23). In this study, we investigated how changes in temperature, culture media, and supplementation with NaCl and glucose can affect motility. *S. pseudintermedius* exhibited varying levels of motility at the tested incubation temperatures (Fig. 4A). After incubation at 35°C, a significant reduction in the motility on semisolid media was observed compared to incubation at 37°C, although the spreading morphology changed consistently, moving toward one of the edges of the plate. However, at 39°C, increased motility was observed compared to 37°C. These findings are similar to what was previously described for *S. aureus*, which displayed higher motility at 40°C and lower motility at 30°C in comparison to incubation at 37°C (6).

Motility using BHI and LB semisolid media was then compared to motility in TSB semisolid media using standard conditions (Fig. 4B). When cultured in the BHI semisolid media, a distinct colony morphology was observed. In TSB, the colony displayed spreading motility with several distinct dendrites at its edges, whereas in BHI, a more uniform spreading with less-defined dendrites was observed. However, no significant differences in the motility level of *S. pseudintermedius* were detected between these two media. In LB semisolid media, differences in spreading morphology were also observed, with fewer dendrites and a significant reduction in *S. pseudintermedius* spreading compared to TSB. These results align with what was previously described for *S. aureus*, which displayed motility in BHI semi-solid media and was not motile in semisolid LB (6). The effects of 1% NaCl and 1% glucose supplementation in the TSB semisolid media were also evaluated regarding the motility of *S. pseudintermedius* (Fig. 4C). An increase in motility was observed with both supplements, with a statistically significant increase observed in motility with the addition of NaCl. While supplementation with glucose was also shown to increase *S. aureus* motility (6), this is the first study to evaluate the impact of NaCl supplementation on motility of *Staphylococcus* species.

### Impact of different growth conditions on *S. schleiferi* motility

**Fig 5:**
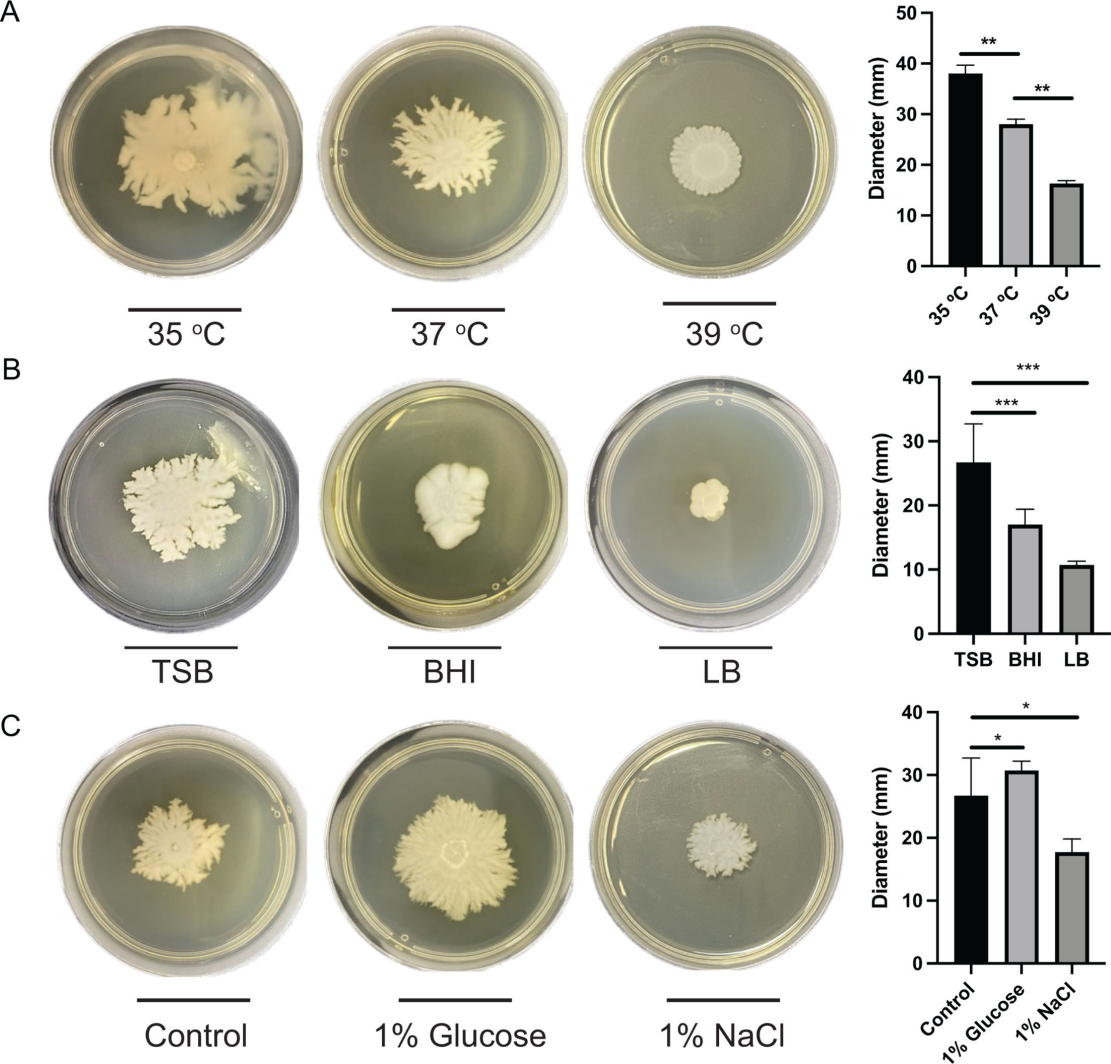
Impact of different growth conditions on the *S. schleiferi* motility. (**A**) Evaluation of the *S. schleiferi* strain 1715 motility in the TSB semisolid media at 35, 37, and 39°C. (**B**) Evaluation of the *S. schleiferi* strain 1715 motility in the TSB, BHI, and LB semisolid media. (**C**) Evaluation of the *S. schleiferi* strain 1715 motility in the TSB semisolid media supplemented with either 1% glucose or 1% NaCl. *: *P* < 0.05; **: *P* < 0.01; ***: *P* < 0.001.

The different conditions mentioned above were also used to investigate if they would similarly affect *S. schleiferi* motility. Noteworthy, the motility of *S. schleiferi* was affected by the different incubation temperatures in a distinct way from what was observed with *S. pseudintermedius* (Fig. 5A). At 35°C, an altered colony morphology was observed, with less dendrites and smoother edges, with a significant increase in the average motility diameter compared to 37°C. On the contrary, at 39°C, a significant reduction in motility was observed, the opposite of what we observed with *S. pseudintermedius*. These findings are also contrary to previous results obtained with *S. aureus* (6). These results highlight the different optimal conditions for movement for each species and may reflect each species' colonization capacity in different hosts, considering that *S. pseudintermedius* is commonly found in dogs, whose average body temperature is 39°C (24), whereas *S. schleiferi*, although also present in dogs, is an opportunistic pathogen in humans who have an average body temperature of 36.5°C (15, 25–27). However, further studies are needed to confirm the relationship between body temperature and the colonization capacity of these species.

When different culture media were evaluated with *S. schleiferi*, a change in the colony morphology was observed in both BHI and LB media, where the colonies showed less-defined dendrites at the edges of the spread compared to TSB (Fig. 5B). Furthermore, the level of motility of *S. schleiferi* was also significantly lower when grown in BHI and LB media compared to TSB. The motility results for *S. schleiferi* in BHI medium differ from what we observed for *S. pseudintermedius* and what was previously described for *S. aureus* (6). In contrast to *S. schleiferi*, both *S. pseudintermedius* and *S. aureus* displayed a level of motility in BHI similar to that in TSB. This once again suggests that the motility pattern of each species is affected uniquely by external conditions. Conversely, *S. schleiferi* motility was completely inhibited in LB semisolid media, registering results comparable to the negative control. This is consistent with findings described for *S. aureus* (6) and with our parallel observations for *S. pseudintermedius*.

Supplementation with 1% NaCl in semisolid TSB resulted in a statistically significant reduction in *S. schleiferi* motility (Fig. 5C). Considering that NaCl concentration in LB (10 g/L) is higher than in TSB or BHI media (5 g/L), the reduction of motility observed with NaCl supplementation might explain the reduction of *S. schleiferi* motility observed in LB. Supplementation of TSB with 1% glucose led to a significant increase in *S. schleiferi* motility (Fig. 5C). These results agree with reports in the literature that described that the addition of glucose and other sugars can stimulate motility in *S. aureus* (10).

Together, these data indicate that growth conditions have a significant impact on *Staphylococcus* motility and that each species responds to changes in a particular way. *S. pseudintermedius* and *S. schleiferi* displayed opposite phenotypes at the different temperatures of incubation as well as after glucose supplementation. However, LB semisolid media was not permissible for motility in either species, while glucose supplementation had a positive impact on both of them.

Understanding the motility mechanisms of *Staphylococcus* species is essential not only from a microbiological perspective but also from a clinical and public health standpoint. Within the One Health concept (28), which argues that animal, environmental, and human health are interconnected, understanding the biology and behavior of pathogens of animal origin, such as the ones in our study, is of utmost importance for human and environmental health.

*S. pseudintermedius* first described in 2005 (29) is considered a commensal of the skin and mucosa of companion animals and can be isolated from up to 90% of healthy dogs (30). It is also an important opportunistic pathogen of dogs, causing external otitis, pyoderma, abscesses, and wound infections in immunosuppressed animals or whenever the skin’s barrier is breached (29, 31, 32). *S. pseudintermedius* is not part of the human microbiota, but humans can act as transient carriers (33). When colonized by *S. pseudintermedius*, human hosts may be asymptomatic or develop serious infections, such as endocarditis, pneumonia, and bacteremia (34–36). The other species investigated in this work, *S. schleiferi*, was first identified in humans in 1988 (37) but has been frequently found in clinically healthy dogs (26). This species can also cause opportunistic infections and can be isolated from pyoderma and otitis in dogs (38, 39). In humans, cases of *S. schleiferi* have been described in clinical syndromes, such as osteomyelitis, endocarditis, pediatric meningitis, surgical wound infections, nosocomial urinary tract infections, and disseminated infections, in immunocompromised patients (15, 27). Both species display increased antimicrobial resistance, and treatment of infections has become increasingly difficult (40, 41). Nevertheless, these veterinary *Staphylococcus* species have been historically understudied despite their veterinary importance and potential for causing zoonotic infections. In this context, this study contributes to the understanding of their biology, focusing on their previously unreported ability to move across a semi-solid surface and determining how different external conditions affect their motility. Further studies are necessary to investigate the molecular mechanisms involved in the motility phenotype of these species.
